# Bilateral Diaphragmatic Nerve Paralysis Secondary to Cervical Spondylosis: A Rare Case

**DOI:** 10.7759/cureus.71904

**Published:** 2024-10-20

**Authors:** Takafumi Kawabata, Kazunori Tobino

**Affiliations:** 1 Respiratory Medicine, Iizuka Hospital, Iizuka, JPN

**Keywords:** bilateral diaphragmatic nerve paralysis, cervical spondylosis, nocturnal dyspnea, noninvasive positive pressure ventilation (nppv), type 2 respiratory failure

## Abstract

Bilateral diaphragmatic nerve paralysis due to cervical spondylosis is an extremely rare condition with only one previously reported case. We present a 64-year-old Japanese male with a history of left diaphragmatic nerve paralysis who developed sudden nocturnal dyspnea. Physical examination revealed orthopnea and type 2 respiratory failure. Imaging studies showed bilateral diaphragmatic elevations and cervical spine stenosis at C3/C4 and C4/C5 levels. Pulmonary function tests demonstrated a significant reduction in vital capacity, particularly in the supine position. After excluding other potential causes, the diagnosis of bilateral diaphragmatic nerve paralysis secondary to cervical spondylosis was established. The patient was successfully treated with noninvasive positive pressure ventilation (NPPV) and showed gradual improvement in symptoms and diaphragmatic function over three years of follow-up. This case highlights the importance of considering cervical spondylosis as a potential etiology for bilateral diaphragmatic nerve paralysis and demonstrates the effectiveness of NPPV in managing this rare condition. Regular monitoring and long-term follow-up are crucial for optimal management of these patients.

## Introduction

Bilateral diaphragmatic nerve paralysis due to cervical spondylosis is an exceedingly rare condition, with only one previously reported case in the literature by Keelan et al. [1]. In this article, we present a case of bilateral diaphragmatic nerve paralysis caused by cervical spondylosis, highlighting the diagnostic challenges associated with this unusual presentation. Cervical spondylosis is a well-recognized cause of unilateral diaphragmatic nerve paralysis, as illustrated in the case report by Manabe et al., where a patient with cervical spondylosis developed unilateral (hemidiaphragmatic) phrenic nerve palsy secondary to C4 radiculopathy [2]. This case demonstrates that cervical spondylosis can indeed affect the phrenic nerve, albeit only unilaterally in this particular instance. The pathophysiological mechanism underlying this phenomenon is thought to be related to the compression of the nerve roots that contribute to the phrenic nerve, leading to focal neuropathy. A review article by Ali et al. provides further evidence of the potential impact of cervical spondylosis on phrenic nerve function [3]. The authors found that patients with cervical spondylosis exhibited prolonged phrenic nerve distal motor latency, suggesting the presence of subtle bilateral phrenic nerve involvement. However, it is important to note that this review did not describe any cases of overt bilateral diaphragmatic nerve paralysis, underscoring the rarity of this presentation. Despite evidence that cervical spondylosis can affect the phrenic nerves and cause unilateral diaphragmatic nerve paralysis, a thorough literature search did not yield any additional documented cases of bilateral diaphragmatic nerve paralysis attributed to cervical spondylosis, apart from the singular case reported by Keelan et al. [1]. This paucity of reported cases highlights the exceptional nature of this clinical entity and the need for increased awareness among healthcare professionals. In this report, we describe a case of bilateral diaphragmatic nerve paralysis secondary to cervical spondylosis that posed significant diagnostic challenges. The atypical presentation and the absence of well-established diagnostic guidelines for this condition contributed to the complexity of the case. Through this article, we aim to shed light on this rare manifestation of cervical spondylosis and provide valuable insights to aid in its recognition and management.

## Case presentation

A 64-year-old Japanese male presented to our emergency department with sudden nocturnal dyspnea while in the supine position. He had been followed at our respiratory department since the age of 61 years due to left diaphragmatic nerve paralysis of unknown cause and was asymptomatic. He was a former smoker with a 60-pack-year smoking history and consumed one beer and three glasses of shochu daily. His initial vital signs were as follows: body temperature, 35.9℃; heart rate, 68 beats per minute; blood pressure, 143/86 mmHg; respiratory rate, 20 breaths per minute; and oxygen saturation, 96% while sitting and 85% in the supine position on room air. Physical examination, including auscultation and cardiovascular and abdominal assessments, was unremarkable. Blood tests revealed a normal complete blood count, chemistry, electrolytes, kidney and liver function, and serum immunoglobulin levels, with an HbA1c of 6.9% (Table 1).

**Table 1:** Results of laboratory tests.

Test	Result	Reference range
White blood cells	4060/μL	3300-8600/μL
Hemoglobin	15 g/dL	13.7-16.8 g/dL
C-Reactive Protein	0.03 mg/dL	0-0.14 mg/dL
HbA1c	6.9	4.9-6.0 %
Carcinoembryonic antigen	2.7 ng/mL	0-5 mg/mL
Cytokeratin 19 fragment	1.8 ng/mL	0-3.5 mg/mL
Gastrin-releasing peptide	45.8 mg/mL	0-81 mg/mL
Epstein-Barr virus (EBV) EBV VCA IgM EBV VCA IgG EBV EA(D) IgG EBNA IgG	Negative Positive Positive Positive	Negative Negative Negative Negative
Hepatitis B virus Hep B surface antigen Hep B surface antibody Hep B core antibody	Non-reactive Non-reactive Positive	Non-reactive Non-reactive Non-reactive
Acetylcholine receptor binding antibody	Negative	Negative
Anti-GM1 monoclonal antibody	Negative	Negative
Anti-voltage-gated calcium channel	Negative	Negative

**Figure 1:**
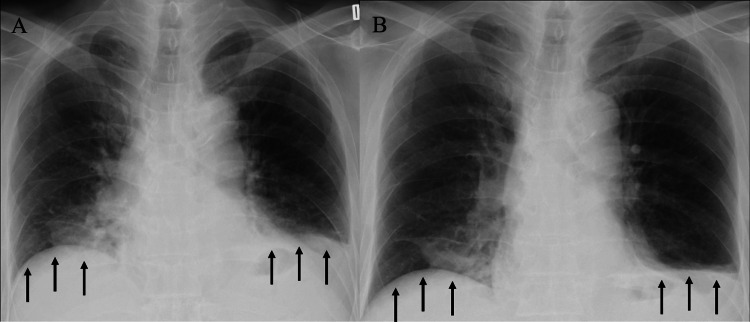
Chest radiographs during inspiration and expiration. Chest radiographs showed no appreciable difference in the diaphragm position between inspiration (A) and expiration (B), suggestive of bilateral diaphragmatic paralysis. The black arrow points to the diaphragm, which has poor movement during respiration.

Blood gas analysis showed a pO2 of 66.4 mmHg, pCO2 of 50.4 mmHg, and type 2 respiratory failure. Chest radiograph showed bilateral diaphragmatic elevations and almost no movement of the diaphragm with respiration (Figure 1).

A chest CT scan demonstrated no active lesions in the lung fields but decreased volumes in the bilateral lower lung lobes. Pulmonary function tests (PFTs) showed a vital capacity (VC) of 2.57 L (61.3% of predicted value) in the sitting position and 1.54 L (36.8% of predicted value) in the supine position, a decrease of more than 20%. The patient had undergone surgery for cholangitis at our hospital three years earlier, and a PFT performed at that time showed a VC of 4.21 L (98.8% of predicted value). Based on these findings, a diagnosis of acute type 2 respiratory failure due to bilateral diaphragmatic nerve paralysis was made.

**Figure 2:**
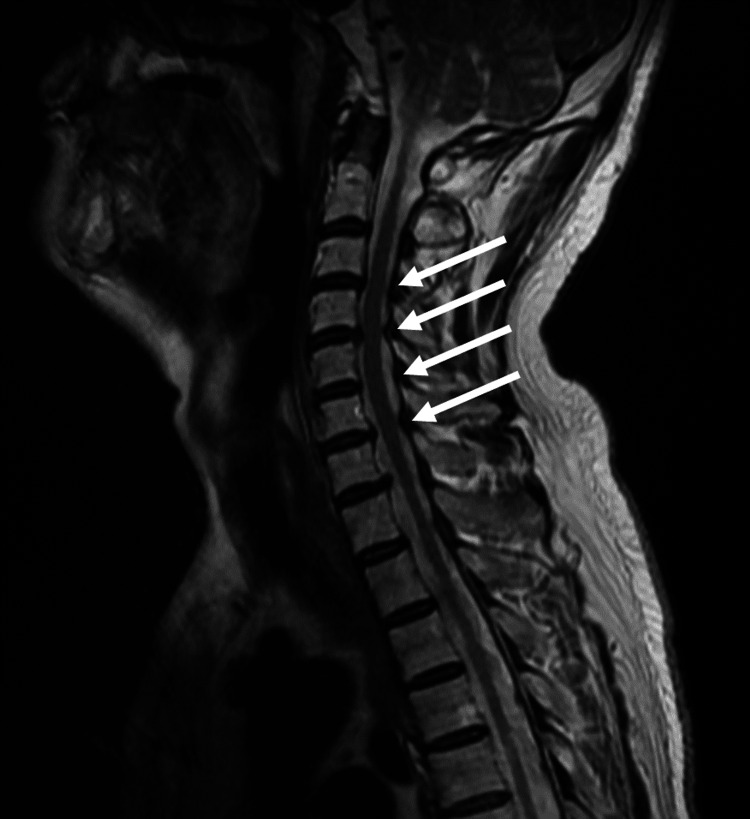
Cervical spine MRI demonstrating cervical spondylosis. Cervical spine MRI scan reveals osteophytes from C3/4 to C7/T1 levels and posterior protrusion of intervertebral discs, causing compression of the dural sac. These findings are consistent with cervical spondylosis.

The patient was admitted to the hospital for further investigation of the cause of the diaphragmatic nerve palsy. Nerve conduction velocity testing and needle electromyography showed no abnormal findings. There was no sudden onset of severe neuralgic pain and muscle weakness with atrophy localized to the upper extremities after the onset of pain, both of which are signs of neuralgic amyotrophy. Autoantibodies for Guillain-Barré syndrome and myasthenia gravis were negative, and infectious diseases such as herpes simplex, varicella-zoster virus, tuberculosis, and polio were considered unlikely based on his medical history and antibody tests. Tumor markers were negative, but mediastinal lymphadenopathy was seen on chest CT, so an ultrasound-guided transbronchial needle aspiration (EBUS-TBNA) was performed. Histological examination of the mediastinal lymph nodes revealed no obvious malignant findings or findings suggestive of sarcoidosis. He had a history of type 2 diabetes mellitus, which could cause neuropathy, but this was not strongly suspected as the primary cause because of good glycemic control. A few days before the visit, he had cervical pain, and a cervical MRI scan showed stenosis at C3/C4 and C4/C5, leading to a diagnosis of cervical radiculopathy (Figure 2).

**Figure 3:**
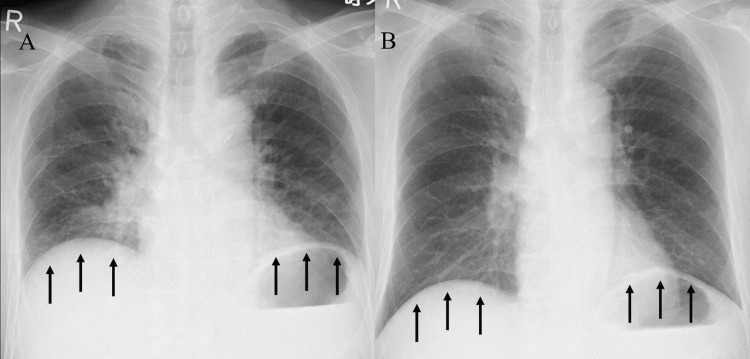
Follow-up chest radiographs showing improved diaphragmatic movement. Follow-up chest radiographs demonstrate a discernible change in diaphragm position between expiration (A) and inspiration (B), indicating improved diaphragmatic function. The black arrow points to the diaphragm, with improved movement during breathing.

Since other diseases causing diaphragmatic nerve paralysis could be ruled out, a diagnosis of bilateral diaphragmatic nerve paralysis secondary to cervical radiculopathy was made. Due to the lack of improvement in orthopnea and type 2 respiratory failure, he was treated with noninvasive positive pressure ventilation (NPPV) at night (nine hours a day), with an inspiratory positive airway pressure (IPAP) of 17 cm H2O and expiratory positive airway pressure (EPAP) of 4 cm H2O. He was able to fall asleep at night promptly after the introduction of NPPV, and his type 2 respiratory failure improved. Home ventilatory management was introduced, and he was discharged home one month after admission. The orthopnea slowly improved, and chest radiographs taken three years later showed improved respiratory movement of the diaphragm (Figure 3).

The PFT performed at that time improved the VC to 3.03 L (73.9% of the predicted value).

## Discussion

In this report, we presented a rare case of bilateral diaphragmatic nerve paralysis secondary to cervical spondylosis, which was successfully managed with NPPV. The patient's initial presentation with sudden nocturnal dyspnea and type 2 respiratory failure, along with a history of left diaphragmatic nerve paralysis of unknown cause, led to a thorough investigation to identify the underlying etiology. After excluding other potential causes, such as neuromuscular disorders, infections, and malignancies, the diagnosis of bilateral diaphragmatic nerve paralysis due to cervical spondylosis was established based on his clinical presentation and imaging findings.

Many etiologies can cause diaphragmatic paralysis, including pneumonia, compressive mediastinal tumors, myelopathy, neuropathy, myopathy, and trauma (including during central venous cannulation and head and neck surgery). However, the etiology of diaphragmatic paralysis has not been identified in two-thirds of cases [4]. Our case adds to the limited literature on bilateral diaphragmatic nerve paralysis caused by cervical spondylosis, with only nine previous cases reported by Keelan et al. [1]. While cervical spondylosis is a well-recognized cause of unilateral diaphragmatic nerve paralysis [2], its association with bilateral diaphragmatic nerve involvement has been rarely described. The review article by Ali et al. [3] suggests that cervical spondylosis may lead to subtle bilateral diaphragmatic nerve paralysis as evidenced by prolonged phrenic nerve distal motor latency. However, the occurrence of overt bilateral diaphragmatic nerve paralysis, as seen in our patient, highlights the potential for cervical spondylosis to cause clinically significant bilateral phrenic nerve impairment.

Treatment of respiratory failure due to bilateral diaphragmatic nerve palsy is suggested to remove the cause, if known [5]. For example, if the cause is cervical cord compression by a tumor, decompression may be effective. On the other hand, diaphragmatic paralysis due to potentially reversible aetiologies (e.g., surgical, tumor-related, diabetic neuropathy) has been shown to improve spontaneously in 40-60% of cases over time, as has diaphragm and respiratory muscle strength [6,7]. Surgical diaphragmoplasty is indicated for unilateral diaphragmatic paralysis but not for bilateral diaphragmatic nerve paralysis. Diaphragmatic pacemakers are another option, but only in limited cases. NPPV is therefore indicated for bilateral diaphragmatic nerve palsy, with some reports of successful treatment [8]. However, in cases of acute respiratory failure, intubation or tracheostomy may be required, and the gradual improvement in the patient's orthopnea and diaphragmatic movements after initiation of NPPV suggests that nerve compression is reversible and that diaphragmatic nerve function may be restored with appropriate management.

## Conclusions

This case highlights a rare instance of bilateral diaphragmatic nerve paralysis secondary to cervical spondylosis, managed successfully with NPPV. It emphasizes the importance of considering cervical spondylosis in the differential diagnosis of unexplained diaphragmatic nerve paralysis, particularly in patients with a history of cervical pain or neuropathy. NPPV proved effective as a treatment modality, leading to gradual improvement in the patient's orthopnea and diaphragmatic function. The case suggests that early diagnosis and appropriate management can result in favorable outcomes. It underscores the need for increased awareness of this rare complication and emphasizes the importance of establishing diagnostic criteria and optimal management strategies.
